# A Global Picture of Molecular Changes Associated to LPS Treatment in THP-1 Derived Human Macrophages by Fourier Transform Infrared Microspectroscopy

**DOI:** 10.3390/ijms232113447

**Published:** 2022-11-03

**Authors:** Diletta Ami, Ana Rita Franco, Valentina Artusa, Paolo Mereghetti, Francesco Peri, Antonino Natalello

**Affiliations:** 1 Department of Biotechnology and Biosciences, University of Milano-Bicocca, Piazza della Scienza 2, 20126 Milan, Italy; 2 Independent Researcher, 15061 Arquata Scrivia, Italy

**Keywords:** biomarkers, inflammation, infrared microspectroscopy, lipopolysaccharide, multivariate analysis, THP-1 derived macrophages

## Abstract

Macrophages are among the first immune cells involved in the initiation of the inflammatory response to protect the host from pathogens. THP-1 derived macrophages (TDM) are used as a model to study the pro-inflammatory effects of lipopolysaccharide (LPS) exposure. Intact TDM cells were analysed by Fourier transform infrared (FTIR) microspectroscopy, supported by multivariate analysis, to obtain a snapshot of the molecular events sparked by LPS stimulation in macrophage-like cells. This spectroscopic analysis enabled the untargeted identification of the most significant spectral components affected by the treatment, ascribable mainly to lipid, protein, and sulfated sugar bands, thus stressing the fundamental role of these classes of molecules in inflammation and in immune response. Our study, therefore, shows that FTIR microspectroscopy enabled the identification of spectroscopic markers of LPS stimulation and has the potential to become a tool to assess those global biochemical changes related to inflammatory and anti-inflammatory stimuli of synthetic and natural immunomodulators different from LPS.

## 1. Introduction

Inflammation is a key host response that ensures protection against injury or infection. In the case of microbial infection, the innate immune system plays a very important role in the initiation of the inflammatory response. It can recognize highly conserved pathogen-associated molecular patterns (PAMPs) through different specialised receptors, thus starting a cascade of pro-inflammatory events in order to protect the host from the pathogen [1]. Among the first lines of cellular defence against infection, macrophages are extremely plastic cells able to rapidly change their functional profile through a process defined as polarisation. Polarisation is the process by which macrophages respond to stimuli coming from the local microenvironment and acquire a specific functional phenotype [2].

Classical activated macrophages, described as the M1 phenotype, are part of this local non-specific response to PAMPs as they are able to produce pro-inflammatory cytokines such as TNF-α, IL-6 and IL-1β, and also have microbicidal activity. Alternatively activated macrophages, M2 phenotype, are characterised by an anti-inflammatory profile which permits resolution of inflammation and tissue repair [3]. One of the PAMPs responsible for M1 activation is lipopolysaccharide (LPS), the major component of the outer leaflet of the gram-negative bacteria outer membrane, which strongly activates Toll-like receptor 4 (TLR4) in different immune cells, including macrophages [4]. LPS has been linked to several diseases including sepsis, acute lung injury, asthma, and atherosclerosis; thus, the signals it elicits in the host cell, although incompletely described, are of great biomedical significance.

The THP-1 monocytic human leukaemia cell line is a validated model to investigate monocyte and macrophage functions, mechanisms, and immune signalling pathways. In order to study the effects of LPS exposure, these cells can be used as monocytes or differentiated into THP-1 derived macrophages (TDM), by exposure to a pro-inflammatory stimulus prior to LPS stimulation [5]. Notably, the capability of LPS to induce gene expression in THP-1 derived macrophages was compared with the LPS-triggered response of human primary, peripheral blood mononuclear cell (PBMC) derived macrophages. The study concluded that although THP-1 cells are transformed and immortalised, their LPS–induced gene expression signature remains very similar to primary macrophages [6]. This model is reliable, simple and suitable for the investigation of inflammation-related macrophage responses [5] and thus it was selected for this study.

After the LPS challenge, the macrophage cell machinery is confronted with different activations that ultimately lead to a cellular phenotype switching. Specifically, TDM stimulation by LPS triggers different pro-inflammatory pathways within the cell through different effector proteins, namely MyD88 and TRIF [7], and also through a cytosolic protein complex known as NLRP3 inflammasome [8]. LPS also induces important remodelling of the cellular metabolism in macrophages, including glycogen metabolism [9] and a strong enhancement of glycolysis [10]. The increase in glycolysis products has the potential to burst the de novo biosynthesis of fatty acids, and to promote the expansion of the endoplasmic reticulum (ER) and Golgi to accommodate the increased demand for the synthesis, transport and secretion of inflammatory cytokines and chemokines. Thus, LPS triggers a complex response within the cell that requires a metabolic reprogramming at the expense of cellular energy provided by the increase in uptake of different molecules such as glucose, amino acids and fatty acids. It is known that lipids and their metabolism play an important role in ensuring macrophage plasticity throughout this process [11].

The aim of this work was to develop and validate a method with the capacity to provide at a glance a global picture depicting the biochemical changes occurring in an in vitro model of inflammation, in our case LPS-stimulated TDM. Specifically, methods able to detect in intact cells changes in protein, lipid and sugar/glycan composition in a non-time-consuming way could be useful to this scope.

In this perspective, Fourier transform infrared (FTIR) microspectroscopy has proven to be a powerful tool to monitor, in a non-destructive and label-free way, the global biochemical composition of whole cells, through the absorption of electromagnetic radiation in the mid infrared range. This vibrational tool probes a large number of molecules simultaneously, thus its sensitivity to structural and compositional changes makes it complementary to other biochemical methods [12,13]. For these reasons, FTIR spectroscopy has become an attractive tool in molecular and cellular biophysics with important applications also in biomedical research [13,14,15,16,17,18,19]. Given the complexity of cell spectra resulting from the overlapping absorptions of cell lipids, proteins, nucleic acids and carbohydrates, their interpretation requires a sophisticated multivariate analysis able to point out significant and non-redundant information [15,18,20].

Here, we describe the use of FTIR microspectroscopy coupled to Partial Least Squares-Discriminant Analysis (PLS-DA) to investigate in situ the biochemical modifications occurring in human TDM cells treated with LPS. The results of the multivariate analysis highlighted the most significant spectral components affected by the treatment, ascribable mainly to lipid and protein bands, thus stressing the fundamental role of these classes of molecules in inflammation and in immune response.

## 2. Results and Discussion

The biochemical modifications occurring in TDM cells exposed to LPS were investigated by FTIR microspectroscopy. In particular, we employed PLS-DA to compare FTIR data obtained from intact TDM treated with LPS at different time points over the course of 24 h, with data obtained from non-treated TDM. This multivariate analysis allowed not only the evaluation of the statistical significance of the observed spectral differences, but also the identification of the spectral components responsible for the discrimination between treated and untreated cells, also taking into account the period of LPS exposure. The analysis has been performed on the second derivative spectra that have been calculated to better resolve the overlapped components in the absorption bands (Figure S1), necessary for the identification of peak positions and their assignment to the vibrational modes of the different molecules [13,18,20].

**Figure 1:**
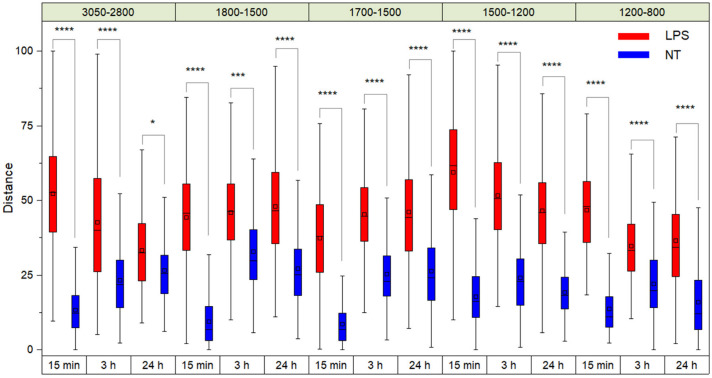
Euclidean distance values of the PLS-DA projections of TDM cells treated with LPS and non-treated cells (NT) for time zero analyses (0 h). The black horizontal line within the box is the median, the square within the box is the mean, the box ends show the first (Q1) and third (Q3) quartiles, the lower whisker is computed as the maximum value between the absolute minimum and Q1 − 1.5 × IQR, and the upper whisker is the minimum between the absolute maximum and Q3 + 1.5 × IQR. Here, IQR is the interquartile range computed as Q3–Q1. Stars (*, ***, ****) indicate the statistical significance evaluated by a two-ways repeated-measurement ANOVA analysis.

Figure 1 shows the Euclidean distance values of the PLS-DA projections of TDM cells treated with LPS and zero-time untreated cells (0 h), considering both the different spectral ranges and the period of incubation with LPS. The two-ways repeated-measurement ANOVA analysis indicates that the distances between treated and untreated TDM cells were significant for all the spectral regions and times considered (Figure 1).

All the above considered, in the following figures, where the mean second derivative spectra are displayed, we will discuss mainly the spectral components responsible for the PLS-DA discrimination between LPS-treated and untreated TDM cells (only the components with overall weight factor ≥75% were considered). In the Supplementary Table S1 the assignment of the peaks identified in the analysed spectral ranges has been reported. To visualize the separation of the LPS treated (24 h) and untreated (0 h and 24 h) cells, the PLS-DA score plots have been reported in the Figure S2. The PLS-DA discrimination performance, evaluated using the classification accuracy, has been shown in the following figures as box-plots.

### 2.1. FTIR Analysis of Protein Secondary Structure Modifications in TDM Cells Exposed to LPS

**Figure 2:**
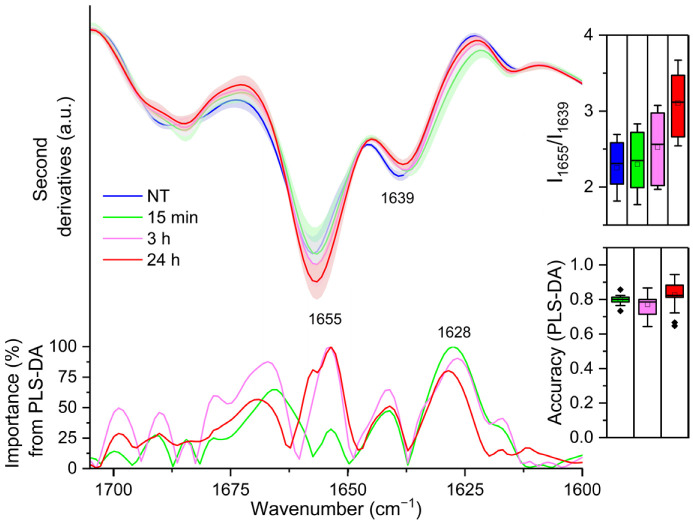
Mean second derivative spectra in the Amide I band of THP-1 cells before (0 h) and at different time points of LPS treatment. Standard deviation has also been displayed as a shadowed area. In the inset, the intensity ratio between the 1655 cm^−1^ and the 1639 cm^−1^ peaks, taken from the second derivatives, is illustrated. Below, the wavenumber importance for PLS-DA discrimination performed in the 1700–1600 cm^−1^ spectral range is shown. The PLS-DA discrimination accuracy has been also reported. Box plots are given as in Figure 1.

Figure 2 reports the second derivative analysis of the Amide I band, in the 1700–1600 cm^−1^ range, due to the C=O stretching vibration of the peptide bond, which provides information on the secondary structures of the whole cell proteins [21,22]. Untreated cells second derivative spectrum (0 h) was characterised by a component at ~1655 cm^−1^, which arises from α-helix and random coil structures and a component at ~1639 cm^−1^, which can be assigned to β-sheets [21,22]. After 15 min of LPS treatment, a downshift of the β-sheet band (to ~1637 cm^−1^) was detected (see also loading plot of Figure 2), accompanied by the appearance of a shoulder at lower wavenumbers that can be assigned to intermolecular β-sheets, typical of protein aggregates and/or protein–protein interactions ([19,23,24] and references cited therein). Then, starting from 3 h of treatment with LPS, and up to the end of our observation (24 h), an increased intensity in the α-helix/random coil band at ~1655 cm^−1^ was found, together with the disappearance of the shoulder at ~1628 cm^−1^ (see the loading plot of Figure 2). Moreover, in particular in the 24 h LPS-treated spectrum, a decreased intensity in the 1638 cm^−1^ band was also detected (see the loading plot of Figure 2). Notably, 15 min after LPS administration, PLS-DA identified the spectral component at ~1628 cm^−1^, typical of protein aggregates and/or protein–protein interactions, as the most relevant for the discrimination (overall weight 100%).

The increase in the α-helix band intensity (see also the inset of Figure 2) that we observed in the 3 h (overall weight 100%) and 24 h (overall weight 100%) spectra, accompanied by a further downshift of the β-sheet band, might be diagnostic of LPS-induced expression of proteins with both α-helix and β-sheet structures.

Overall, our findings indicate that LPS stimulation induces significant global changes in the protein content, and likely also in the protein–protein interactions that in the IR spectra can be detected as changes in the whole cell protein secondary structures.

These results are in agreement with Meijer et al. [25] proteomic analysis that showed that LPS stimulation leads to significant up-regulation of a cluster of pro-inflammatory proteins, including the pro-inflammatory proteins macrophage migration inhibitory factor (MIF) and TNF-α, and various pro-inflammatory chemokines. The increased expression of several chemokines points to an important role of LPS-stimulated macrophages in recruiting other immune cells to sites of inflammation. Another upregulated functional cluster consists of proteins involved in actin cytoskeleton organisation, migration, chemotaxis and phagocytosis, as well as in transduction of LPS-induced TLR4 signalling and MHC-II mediated antigen presentation [25]. Proteoglycans (PGs)—glycosylated proteins, which have covalently attached glycosaminoglycans (GAGs)—are also known to be produced as extracellular and cell surface proteoglycans in response to LPS stimulation [26]. Moreover, Dhungana et al. [27] reported a stimulus-induced statistically significant global increase in the number of distinct proteins in rafts in LPS-stimulated RAW macrophages. Authors described an LPS-induced dynamic exchange of proteasome subunits in macrophage triggered by LPS, which is thought to be “targeted” to lipid rafts in host cells in part through its interactions with raft-resident proteins (i.e., CD14). Specifically, they suggest that during LPS exposure a substantial number of new (i.e., basally raft-excluded) proteins are recruited to rafts in a time-dependent manner. Our observations about protein–protein interactions could pair with their data that collectively provide evidence for the localization of proteasome subunits to rafts, their reorganisation within rafts following LPS exposure, and an associated functional change in proteasomal activity specific to rafts that occurs during LPS signalling [27].

Interestingly, the appearance of the 1628 cm^−1^ shoulder might be associated with the interaction of LPS with the TLR4/MD-2 complex situated on the plasma membrane to initiate the MyD88-dependent signalling. It has been reported that the activation of this pathway involves the formation of an intracellular oligomeric signalling complex called the myddosome, formed by MyD88 and members of the IRAK family [28]. Furthermore, another supramolecular organising centre (SMOC) can be attributed to LPS signalling through TLR4, which is the triffosome. LPS bound to TLR4/MD-2 and CD14 receptors undergoes internalisation in the endosome where it triggers the TRIF-dependent through the assembly of a complex of TRAM and TRIF [29].

### 2.2. FTIR Analysis of Lipid Modifications in TDM Cells upon Exposure to LPS: Insights from the 1500–1200 cm^−1^ and 3050–2800 cm^−1^ Spectral Ranges

**Figure 3:**
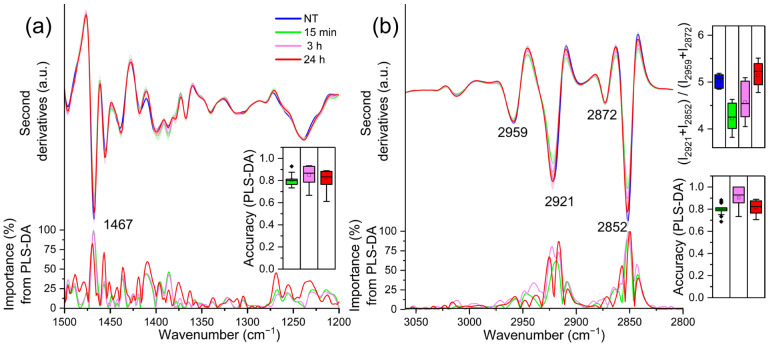
Mean second derivative spectra in the 1500–1200 cm^−1^ (**a**) and 3050–2800 cm^−1^ (**b**) ranges of TDM cells before (0 h) and at different time points of LPS treatment. Standard deviation has also been displayed as a shadowed area. Below, the wavenumber importance for PLS-DA discrimination performed in the selected spectral range is shown for each panel. In the insets, the PLS-DA discrimination accuracy (panels (**a**,**b**)) and the intensity ratio among the CH_2_ and the CH_3_ peaks (panel (**b**)) are reported. Box plots are given as in Figure 1.

The infrared absorption of TDM cells in the 1500–1200 cm^−1^ spectral range is mainly due to the absorption of methyl and methylene groups from different biomolecules, including lipid hydrocarbon chains and head groups [22,30,31]. Moreover, the absorption of phosphate groups mainly from nucleic acids and lipids also occurs in this spectral range, as indicated by the broad band at ~ 1240 cm^−1^, which is assigned to the antisymmetric phosphate (PO2-) stretching mode [22,30,31,32]. Notably, comparing the NT (0 h) sample with LPS-treated cells spectra, PLS-DA identified as relevant—at each time point—only the component at ~1467 cm^−1^ (Figure 3a), mainly ascribable to the overlapping absorption of CH_2_ and CH_3_ groups from lipid hydrocarbon chains [22,30,31]. The intensity of this component, higher in the NT (0 h) cells, decreases rapidly at 15 min, and then increases again up to 24 h.

To investigate more in detail the effects of LPS on THP-1 derived macrophage lipids, FTIR analysis has been extended to the 3050–2800 cm^−1^ spectral range, dominated by the absorption of lipid hydrocarbon chains. As shown in Figure 3b, the spectrum of untreated cells (0 h) was characterised by four main absorption bands: ~2921 cm^−1^ and 2852 cm^−1^, due to the antisymmetric and symmetric stretching of CH_2_; the ~2959 cm^−1^ and 2872 cm^−1^, due to the asymmetric and symmetric stretching of CH_3_ [22,30,31].

The CH_2_ bands decreased in intensity at 15 min of LPS treatment, and then increased again at 3 h, up to 24 h. In the inset of the Figure 3a, the ratio between the intensity of the CH_2_ and CH_3_ bands [33,34] is shown. The increase in this ratio, which is significant at 24 h post LPS treatment, is ascribable to the formation of longer hydrocarbon chains, in agreement with the spectral profile variation in the ~1467 cm^−1^ band discussed above (see Figure 3a). In addition, this result is also supported by the spectral changes displayed by the C=O band at ~1742 cm^−1^, whose intensity, compared to that of untreated cells (0 h), decreased at 15 min and then increased again up to 24 h (see the insert of Figure S3). This absorption is mainly associated with the stretching vibrations of lipid ester groups [22,30,31].

As will be further discussed in the next section (see also Table S1), these results indicate that LPS stimulation induced a modification of the physicochemical properties of cell lipids. Interestingly, recent discoveries point to a complex metabolic network during macrophage activation, particularly regarding macrophage immunometabolism from the perspective of the metabolism of lipids (reviewed by Batista-Gonzalez et al. [35]). In particular, it has been reported that the cell membrane lipid constituents, including glycerophospholipids and sphingolipids, are essential in modulating the pathogen recognition, and alterations in these molecules have been reported in LPS stimulated cells [11,36]. Among these, it has been speculated that some sphingolipids could have a crucial role in the regulation of the early pro-inflammatory or late pro-resolution phases of TLR4 activation [37]. Furthermore, phospholipids play a crucial role in cell-mediated inflammatory responses, including LPS-induced inflammation, since they are important signalling molecules involved in the regulation of cytokine production and because they provide precursors for the synthesis of potent lipid mediators [38]. Recent experimental evidence suggests that LPS-stimulated macrophages increase their fatty acid synthesis to store increased amounts of triacylglycerols and cholesterol esters in lipid droplets [39]. This synthesis appears to be required for T-cell priming [40], inflammasome activation [41] and pro-inflammatory cytokine production [39,40].

### 2.3. FTIR Analysis of TDM Exposed to LPS: Analysis of the Fingerprint Region

The 1200–800 cm^−1^ spectral range, the so-called fingerprint, is a very crowded and highly overlapped region of the spectrum, being dominated by the absorption of complex carbohydrate modes, isolated and/or associated or bound to other molecules, with important contributions also from lipids and nucleic acids. For this reason, the band assignment in this spectral range is not easy, and sometimes, not unequivocal. Overall, as we will describe below, the spectral components pulled out by PLS-DA are mostly ascribable to saccharides (including also GAGs) and lipids, in particular phospholipids and sphingomyelin (SM, Table S1).

**Figure 4:**
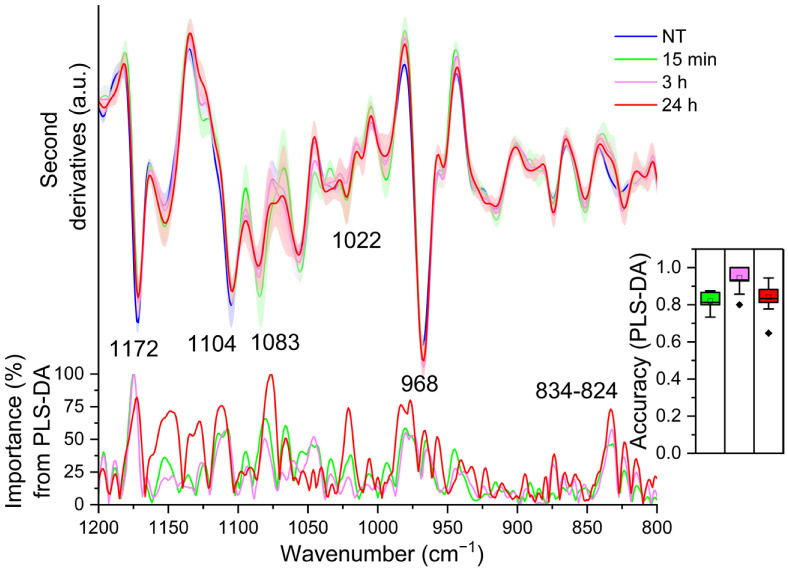
Mean second derivative spectra in the 1200–800 cm^−1^ range of TDM cells before (0 h) and at different time points of LPS treatment. Standard deviation has also been displayed as a shadowed area. Below, the wavenumber importance for PLS-DA discrimination performed in the 1200–800 cm^−1^ spectral range is shown. The PLS-DA discrimination accuracy has been also reported in the inset. Box plot is given as in Figure 1.

Examining the loading plot relative to 15 min and 3 h of LPS treatment, PLS-DA brought out only one component carrying the higher spectral variance between treated and untreated cells: the ~1172 cm^−1^ (overall weight ~100) (see the loading plot of Figure 4), which decreased in intensity at 15 min upon LPS incubation, and then increased again up to 3 h (see Figure 4). As mentioned above, the assignment of this band is not immediate due to the overlapping contributions of different components. Considering its intensity variation, which is synchronous with that of other bands assigned to lipid moieties, we tentatively assign it mainly to the CO-O-C stretching vibrations of phospholipids [22,30]. Moreover, it has been also associated with the C-OH and C-C stretching and C-O-H bending of carbohydrates [42,43] and with the SO_4_ and C-O-S stretching of glycosaminoglycans (GAGs), such as heparin and heparan sulphate [44,45]. GAGs are highly heterogeneous linear macromolecules made up of a repeating disaccharide unit, with a variable number of sulphates. They are not only typical extracellular matrix components, but are also present in the cell membrane and as intracellular granules [46]. Considering that GAGs associated with proteoglycans (PGs) are known to play a crucial role in immune defence, being also involved in cytokine and chemokine regulation [47], we cannot exclude also a contribution of these complex molecules to the ~1172 cm^−1^ absorption.

Noteworthy, PLS-DA relative to 24 h of treatment (see the loading plot of Figure 4) depicts a more complex scenario. The analysis, indeed, disclosed different components responsible for the classification: in addition to the ~1172 cm^−1^ band (overall weight ~75%), displaying again a lower intensity compared to NT cells but similar to that of LPS-3 h, PLS-DA identified two more components that occurred at higher intensities in the 24 h treated cells compared to untreated cells (0 h): at ~1022 cm^−1^ and ~968 cm^−1^ (both with an overall weight ~75%). The first band is ascribable mainly to the ring vibrations and stretching vibrations of C-OH from side groups and C-O-C from glycosidic bonds of polysaccharides [42,43], being also typical of glycosylated proteins [48] and lipids [49]. The ~1022 cm^−1^ band falls in a spectral range associated also with the vibrations of GAG pyranose ring structures [44]. Then, considering again the simultaneous variation in intensity of other absorption bands associated with lipids, we assign the component at ~968 cm^−1^ mainly to the asymmetric stretching of the N(CH_3_)_3_ group [30], which is characteristic not only of phosphatidylcholine (PC) but also of the sphingolipid sphingomyelin. In particular, sphingolipids do not just form the building blocks of eukaryotic cell membranes, but they also play a significant role in regulating cell functions. In this regard, Olona et al. reported that LPS induces the biosynthesis of sphingolipids that promote TLR4 signalling in macrophages [37]. We should add that we cannot exclude also a contribution of the C-C stretching of the DNA backbone and/or of RNA ribose-phosphate main chain vibrations to the ~968 cm^−1^ absorption [32].

Furthermore, PLS-DA identified two other significant components that displayed a higher intensity in untreated cells (0 h) compared to 24 h: the component around 1104 cm^−1^ (overall weight ~75%) and that at ~1083–1073 cm^−1^ (overall weight ~100%). The assignment of these bands is particularly difficult, because in this spectral window the absorption of phosphates, mainly from nucleic acids and lipids [30,32], is dominant as well as that of polysaccharides [50]. Indeed, these absorptions are also associated with different vibrational modes from polysaccharide rings, including the ones from GAGs, such as hyaluronic acid [44,51]. Notably, the ~1083–1073 cm^−1^ band is also assigned to the symmetric phosphate (PO2-) stretching mode typical, for instance, of phospholipids and sphingomyelin [30].

Even if its overall weight is lower than 75% (~73%), we should also mention the ~834 cm^−1^ component detected in untreated cells (0 h) and downshifted to ~824 cm^−1^ in 24 h treated cells, where it is also more resolved, which might reflect structural differences in the glycosidic linkages of polysaccharides by being assigned mainly to C1-H ring vibrations [43,52]. Moreover, this absorption has been also assigned to the C-O-S vibrations from GAGs [53,54].

Overall, the fingerprint analysis on one hand supports the importance of the physicochemical variations in lipids triggered by LPS, as discussed above; on the other, it sheds light on a possible involvement of glycan modifications that could impact on the cell mechanical properties, which in turn may contribute to higher phagocytosis activity [55]. In addition, the observed changes, upon LPS stimulation, in sulfated sugars could be associated with the biosynthesis of sulfated glycosphingolipids or GAGs that are known to be endogenous ligands for the TLR4/MD-2 complex [56].

## 3. Materials and Methods

### 3.1. Cell Maintenance

THP-1 X-Blue™ cells (InvivoGen, San Diego, CA, USA) were cultured in RPMI 1640, 2 mM L-glutamine, 10% heat-inactivated foetal bovine serum and 100 U/mL-100 μg/mL of Penicillin-Streptomycin and maintained at 37 °C, 5% CO_2_, 95% humidity. Cells were kept at a density of 0.5 × 10^6^ cells/mL by splitting them every three days. Media and supplements were purchased from Euroclone (Pero, Italy).

### 3.2. Cell Differentiation

THP-1 X-Blue™ monocytes were seeded in 100 mm cell culture dishes (Corning, Borre, France), 5 × 10^6^ cells per dish in 10 mL of medium, and differentiated to TDM by exposure to 100 ng/mL of phorbol 12-myristate 13-acetate (PMA, InvivoGen, San Diego, CA, USA) for 3 days, as previously described [57,58]. Monocyte-to-macrophage differentiation was assessed by optical microscopy inspection. Macrophage-like cells adhered to the support and displayed a flattened and elongated morphology compared to floating round-shaped monocytes. Following differentiation, the PMA containing medium was removed and replaced by PMA-free fresh medium immediately prior to treatment.

### 3.3. Cell Stimulation and Treatments

TDM were stimulated with 100 ng/mL of *Escherichia coli* 055:B5 LPS (Sigma-Aldrich, St Louis, USA) throughout different exposure periods, 15 min, 3 h and 24 h. After exposure to LPS, dishes were placed on ice and the medium removed. Cells were then washed with PBS (Euroclone), scraped using a cell scraper and collected into centrifuge tubes. After centrifugation at 4 °C for 10 min at approximately 125× *g*, PBS was discarded and cell pellets were resuspended in physiological solution (NaCl 0.9%) for further centrifugation at 4 °C, 5 min at 125× *g*. This washing step was repeated 3 times to ensure no medium contamination. Afterwards live cells were resuspended in 10 µL of physiological solution immediately prior to FTIR measurements.

### 3.4. FTIR Microspectroscopy Analysis of Intact THP-1 Derived Macrophage-Like Cells

About 3 μL of cell suspension were deposited onto a BaF_2_ window, transparent to IR, and dried at room temperature for at least 30 min to eliminate the excess of water. FTIR absorption spectra were acquired in transmission mode, in the 4000–700 cm^−1^ spectral range, by a Varian 610-IR infrared microscope coupled to the Varian 670-IR FTIR spectrometer (both from Varian Australia Pty Ltd., Mulgrave VIC, Australia), equipped with a mercury cadmium telluride nitrogen-cooled detector. The variable microscope aperture was adjusted to ~100 μm × 100 μm (spatial resolution). Measurements were performed at 2.0 cm^−1^ spectral resolution; 25 KHz scan speed, triangular apodization, and by the accumulation of 512 scan co-additions. Then, spectra were normalised at the Amide I band area for comparison (Figure S1) and the second derivative analysis was performed (after a 13-point smoothing of the measured spectra) by the Savitzky–Golay method (3rd polynomial, 9 smoothing points), using the GRAMS/32 software (Galactic Ind. Corp., Salem, NH, USA). Absolute peak intensities cannot be obtained from second derivative spectra, where peak height is proportional to the original height and inversely proportional to the square of the original half-width at half-height. However, under the condition that the original absorption spectra are of high quality, second derivatives can be used to monitor spectral changes if the very same analyses are performed on the original data [59,60].

For each sample, we collected several spectra by selecting different areas on the same sample through the variable diaphragm aperture of the infrared microscope. Furthermore, to evaluate the reproducibility of the results, we performed at least three independent experiments.

### 3.5. Multivariate Analysis

Multivariate analysis has been performed using R version 3.6.3. FTIR spectra have been split into five spectral regions and partial least square discriminant analysis (PLS-DA) has been applied on each region, as previously described [34].

PLS-DA is a widely used multidimensional linear regression method, which is a variant of the classical partial least square method when the dependent variable is categorical [61]. To assess the predictive discrimination and avoid over-fitting, for each method a 3-time repeated 5-fold cross-validation was applied; so, for each method, 15 models were trained. Since each sample has multiple spectra, folds have been created at the sample level, ensuring that all spectra for a given sample are either in the training or in the test set. More specifically, having N samples each with m__N_ spectra, on every round of cross-validation, the samples have been partitioned into 5 folds. Four folds (containing N*4/5 samples) have been used to train the model, and the remaining fold (containing N*1/5 samples) was used to test the model. Folds are complementary (i.e., no repeated samples in different folds) and the samples are randomly chosen. The training of the model is repeated 5 times, each time varying the test partition. The 5-fold cross-validation is then repeated 3-times to lower the risk of partition-dependent artefacts. The best model has been selected using the “one standard error rule”. In this case, the model with the best performance value is identified, and using resampling, we can estimate the standard error of performance. The final model used was the simplest model within one standard error of the (empirically) best model [62]. As a performance measure the root mean square error (RMSE) was used. For the PLS-DA method the variable importance measure here is based on weighted sums of the absolute regression coefficients [62]. Each PLS-DA model includes the following 3 classes: NT-T 0 h, NT-Tn, LPS-Tn. Different models have been created for each time: 15 min, 3 h, and 24 h. The discrimination accuracy among the classes was evaluated using the classification accuracy, e.g., the proportion of true results (true positive + true negative) over the total number of samples.

The distribution of distances between TDM cells treated with LPS and untreated has been obtained by computing the Euclidean distance between all pairs of spectra (in the low dimensional PLS score space), between group T 0 h and Tn for NT and for LPS, that is:

$$D{\left(K\right)}_{i,j}=\frac{1}{L}\sqrt{\overset{C}{\underset{c=1}{\sum }}{\left(x_{i}^{c}-x_{j}^{c}\right)}^{2}}$$

where *i* is the *i*-th spectra belonging to the initial time (T 0 h) group, while *j* is the *j*-th spectra belonging to the Tn (where n = 15 min, 3 h, 24 h) group. *L* is the number of PLS scores, and *c* is the *C*-th PLS component. *K* is either NT or LPS. In order to assess the statistical significance of the difference between the distances in NT and LPS and in the time factor, a two-ways repeated measurement ANOVA has been performed.

## 4. Conclusions

The state-of-the-art understanding of LPS signalling is almost certainly incomplete and oversimplified due to the high complexity of the triggered events. The main aim of this work was to obtain a snapshot of the molecular events sparked by LPS stimulation in macrophage-like cells and dissect the molecular components specific of the inflammatory state and of the initiation of the innate immunity signalling in the cell through FTIR microspectroscopy supported by PLS-DA.

The FTIR analysis of intact macrophage cells, a non-destructive, non-time consuming and label-free method to measure the main molecular changes occurring in cells, proved to be the appropriate strategy to achieve our goal. Although FTIR analyses of other inflammation models have been published [63,64], this is, to the best of our knowledge, the first report in which FTIR is applied to study the complex molecular effects caused by LPS stimulation in human macrophages.

Within our global picture of inflammation in TDM, main findings are represented by the identification of different classes of molecules that stand out as the most affected by LPS stimulation. First, the changes in cell protein secondary structures, reflecting changes in protein content and interactions, as the time course evolves, might agree with cell phenomena related to pathway activation, cytokine production and also to proteasome targeted to lipid rafts on the host cell membrane, as described. Second, the significant intensity variation of a few bands ascribable to lipid moieties emphasises their role in the modulation of innate immune response and, therefore, the importance of some lipid molecules as markers of the inflammatory response [37,38]. Notably, the association of lipid metabolism and content inside the cell is a subject that has been far less described than inflammation-related proteins. Indeed, we observed significant differences in the lipid content during 24 h of LPS stimulation, which is congruent with recent research that correlates lipid metabolism and inflammation, showing that lipids can also be a reliable biomarker of inflammation.

Finally, the variations of the IR fingerprint spectral profile upon LPS stimulation also suggest a change in sulfated sugars that could be associated with the biosynthesis of sulfated glycosphingolipids and/or GAGs. In this regard, recent data show that glycosphingolipid sulphates are endogenous ligands for the TLR4/MD-2 complex [56]. Moreover, GAGs play a crucial role in the recruitment and control of a wide range of innate/cellular immune system regulatory proteins [47,65].

In conclusion, our study enabled the untargeted identification of IR spectroscopic markers outlining the cellular effects of LPS stimulation in human macrophages. The establishment of a combination of distinct features, in the form of specific marker bands that describe the initiation of the inflammatory response, has the potential to become a tool to assess, within a single analysis, those global biochemical changes related to an inflammatory stimulus, which can be another synthetic or natural pro-inflammatory TLR4 agonist, different than LPS. Moreover, with the same approach, it could also be possible to perform analyses to evaluate the anti-inflammatory potential of synthetic and natural immunomodulators. Furthermore, our time course approach paired with the assignment of these spectroscopic markers to specific biomolecule classes allowed us to obtain information about the timing of the molecular events that occur after LPS administration. In addition, one of the advantages of our FTIR-based approach is the possibility to identify a priori specific classes of biomolecules associated with a biological event that could be the subject of further investigation, thus aiding basic research, decreasing time and resource consumption. Indeed, the strength of our approach is the possibility of obtaining an integrated view of functional changes using an untargeted analysis instead of focusing on a small set of markers or functions.

Although technological advances are needed to make the proposed FTIR method a large-scale screening tool, our study shows that FTIR microspectroscopy actually provides a broad view of the molecular events associated with LPS stimulation of macrophages and is able to detect those markers that are specific of the initiation of the cell-mediated immune response.

## Data Availability

The data that support the findings of this study are available from the corresponding author upon reasonable request.

## Supplementary Materials

The following supporting information can be downloaded at: https://www.mdpi.com/article/10.3390/ijms232113447/s1.
