# Supplementary material for: A Global Picture of Molecular Changes Associated to LPS Treatment in THP-1 Derived Human Macrophages by Fourier Transform Infrared Microspectroscopy
Source: Int J Mol Sci. 2022 Nov 3;23(21):13447. doi: 10.3390/ijms232113447 (PMC9656053; doi:10.3390/ijms232113447)
Supplement: Supplementary file 1 [file ijms-23-13447-s001.zip › ijms-1983587-supplementary.pdf]

International Journal of
*Molecular Sciences*

MDPI

*Supporting information for*

A global picture of molecular changes associated to LPS treatment in THP-1 derived human macrophages by Fourier transform infrared microspectroscopy

Diletta Ami[1], Ana Rita Franco[1], Valentina Artusa[1], Paolo Mereghetti[2], Francesco Peri[1], Antonino Natalello*[1]

[1] Department of Biotechnology and Biosciences, University of Milano-Bicocca, Piazza della Scienza 2 - 20126 Milan, Italy;

[2] Independent Researcher, 15061 Arquata Scrivia, Italy

*Corresponding Author:

Antonino Natalello, Department of Biotechnology and Biosciences, University of Milano-Bicocca, Piazza della Scienza 2 - 20126 Milan, Italy, e-mail: antonino.natalello@unimib.it

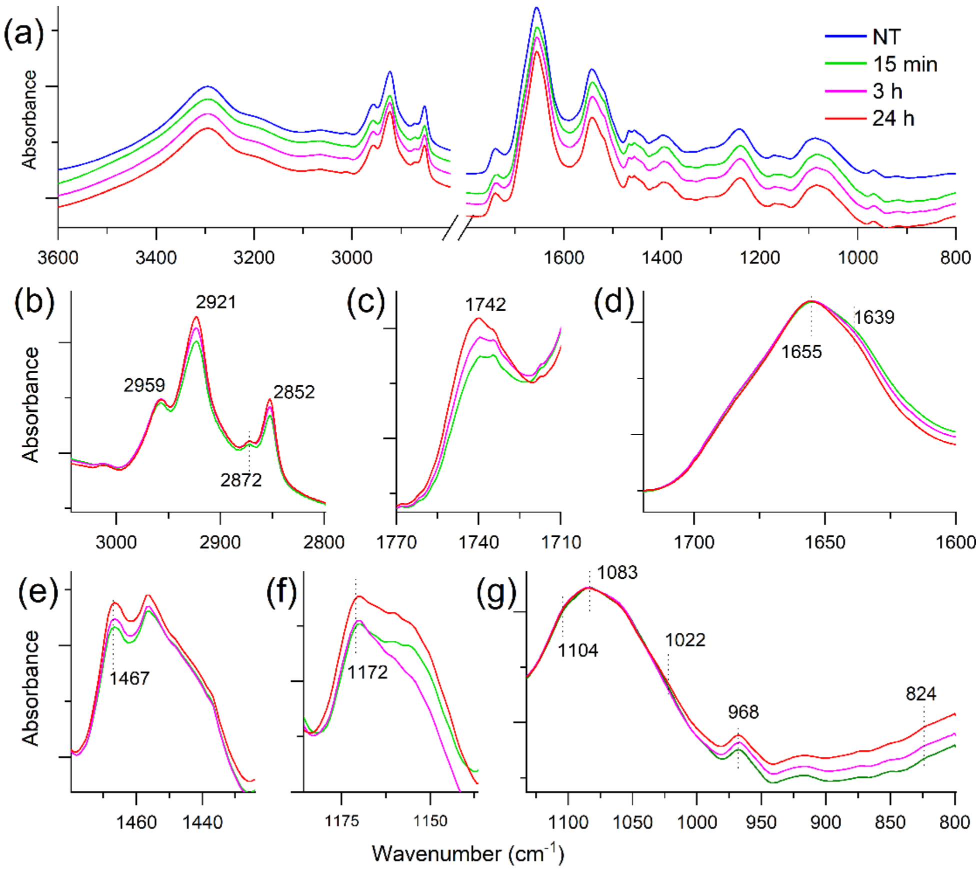


**Figure S1.** Mean absorption spectra for each experimental group. (a) Mean absorption spectra of non-treated (NT) and LPS-treated cells at different time points (15 min, 3 h, 24 h) are displayed in the whole measured range. (b-g) Mean absorption spectra of LPS-treated cells at different time points in selected spectral ranges. The average absorption spectra are reported after normalization at the Amide I band area of the measured spectra, therefore, the absorbance is in arbitrary units and spectra have been offset for the clarity of the figures (a-g).

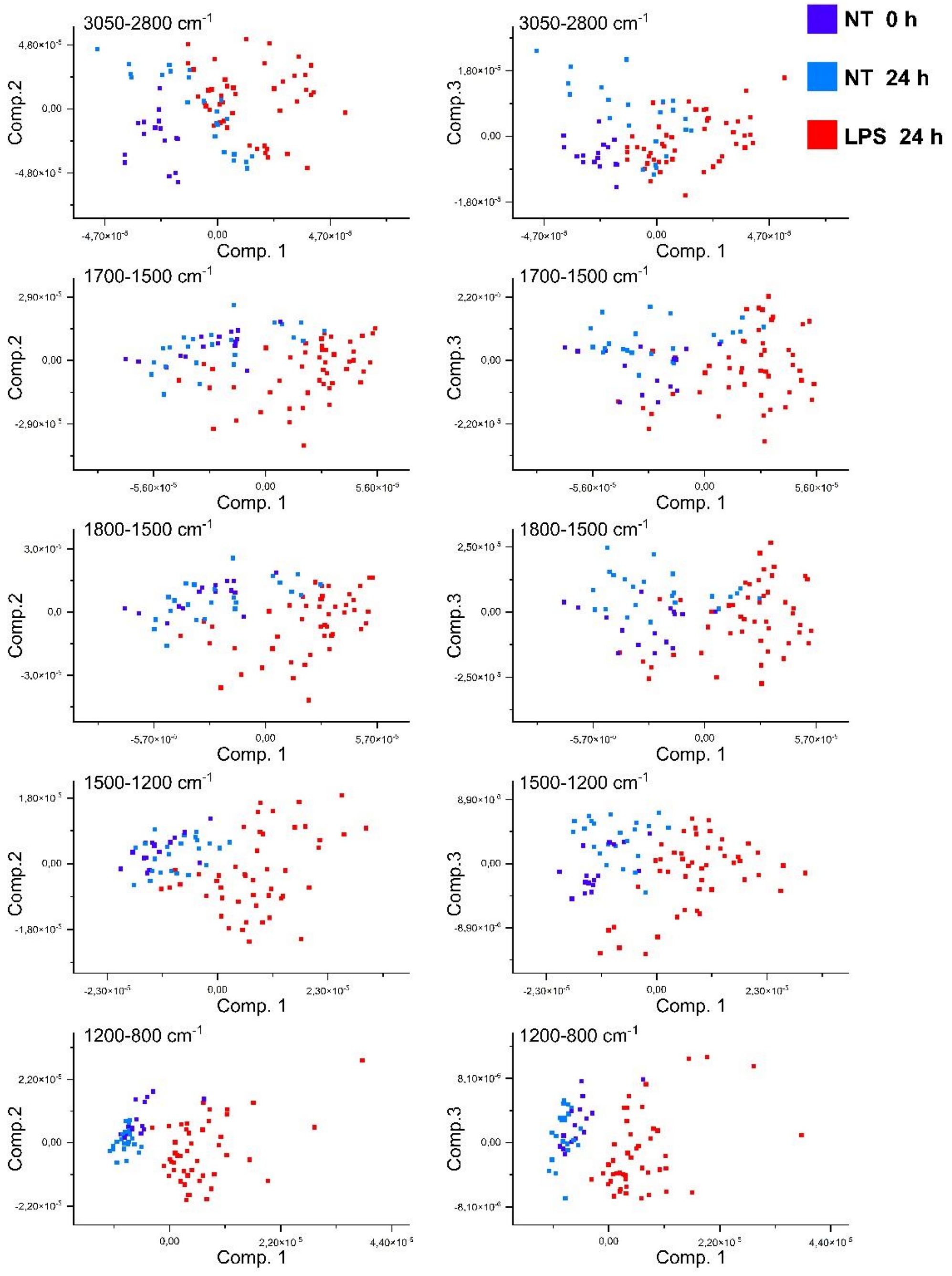


**Figure S2.** PLS-DA score plots. The score plots of Component 2 versus Component 1 and of Component 3 versus Component 1 were reported for the PLS-DA analysis of non-treated TDM cells at 0 h and 24 h and LPS treated TDM cells for 24 h. The PLS-DA analysis was performed in the indicated spectral ranges.

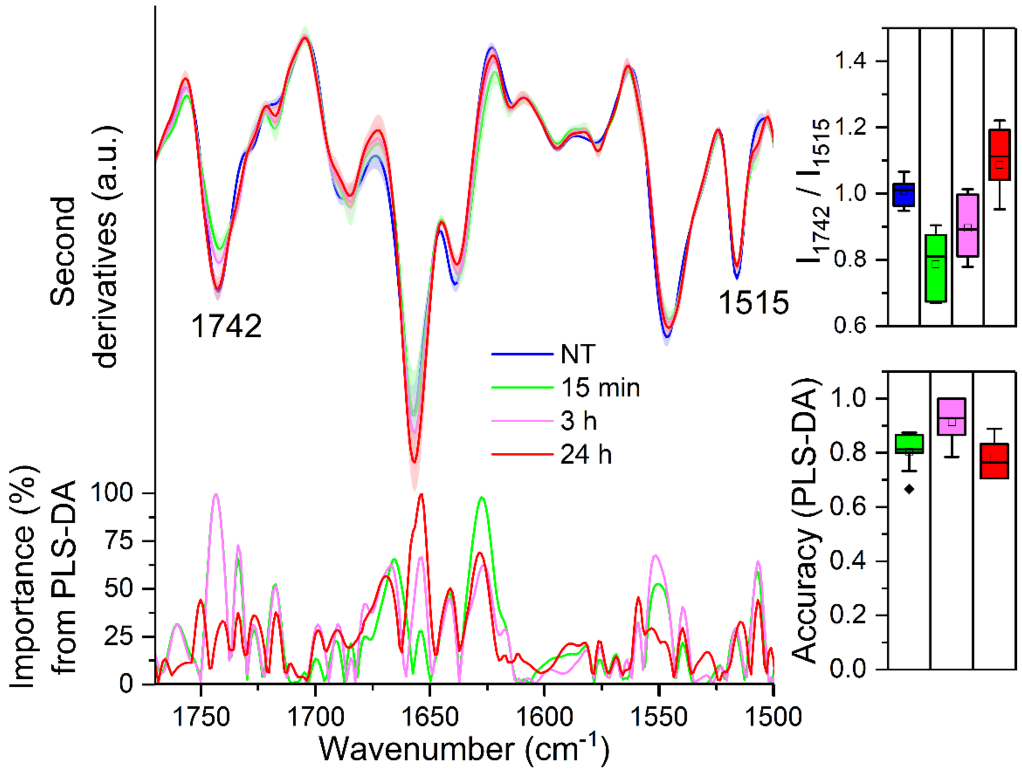


**Figure S3.** Mean second derivative spectra in the 1760-1500 cm$^{-1}$ range of TDM cells before (NT) and at different time points after LPS administration. Standard deviation has also been displayed as a shadowed area. In the inset, the intensity ratio between the C=O band at ~ 1742 cm$^{-1}$ and the tyrosine peak at ~ 1515 cm$^{-1}$ is illustrated. Below, the wavenumber importance for PLS-DA discrimination performed in the 1800-1500 cm$^{-1}$ spectral range is shown. The PLS-DA discrimination accuracy has been also reported. Box plots are given as in Fig. 1.

| Peak positions ($cm^{-1}$) | Assignment | References |
|---|---|---|
| ~2921 | $CH_2$ antisymmetric stretching | [1–3] |
| ~2852 | $CH_2$ symmetric stretching | [1–3] |
| ~1742 | C=O stretching from lipid ester groups | [1–3] |
| ~1655 | α-helices/random coils | [1,4] |
| ~1639-1628 | β-sheets | [1,4] |
| ~1467 | $CH_2$ and $CH_3$ bending, mainly from lipid hydrocarbon chains | [1–3] |
| ~1172 | CO-O-C stretching, mainly from phospholipids;<br>carbohydrates: C-OH, C-C stretching and C-O-H bending;<br>$SO_4$, C-O-S stretching mainly from GAGs | [1,2,5–8] |
| ~1104 | Polysaccharide ring vibrational modes; GAG ring vibrations. | [7,9] |
| ~1083-1073 | Polysaccharide ring vibrational modes;<br>GAG ring vibrations;<br>P=O symmetric stretching $PO^{2-}$ mainly from phospholipids and SM | [1–3,7,9] |
| ~1022 | Polysaccharides: mainly ring vibrations and stretching vibrations of C-OH of side grups and C-O-C of glycosidic bonds;<br>GAG pyranose ring | [5–7,10,11] |
| ~968 | $N(CH_3)_3$ asymmetric stretching of PC and SM;<br>C-C stretching of the DNA backbone and/or of RNA ribose-phosphate main chain vibrations | [2,12] |
| ~834-824 | Glycosidic linkages of polysaccharides (mainly C1-H ring);<br>C-O-S from GAGs | [6,13–15] |

**Table S1:** Assignment of the relevant IR components. The peak positions from second derivative spectra have been reported for the spectral components identified by PLS-DA. The main assignment to the cell biomolecules has been indicated.

**References**


1. Tamm, L.K.; Tatulian, S.A. Infrared Spectroscopy of Proteins and Peptides in Lipid Bilayers. *Q. Rev. Biophys.* **1997**, *30*, 365–429, doi:10.1017/S0033583597003375.
2. Casal, H.L.; Mantsch, H.H. Polymorphic Phase Behaviour of Phospholipid Membranes Studied by Infrared Spectroscopy. *BBA - Rev. Biomembr.* **1984**, *779*, 381–401, doi:10.1016/0304-4157(84)90017-0.
3. Lewis, R.N.A.H.; McElhaney, R.N. Fourier Transform Infrared Spectroscopy in the Study of Lipid Phase Transitions in Model and Biological Membranes. Practical Considerations. In; Dopico, A.M., Ed.; Humana Press: Totowa, NJ, 2007; pp. 207–226 ISBN 978-1-59745-519-0.
4. Barth, A. Infrared Spectroscopy of Proteins. *Biochim. Biophys. Acta-Bioenerg.* **2007**, *1767*, 1073–1101, doi:10.1016/j.bbabio.2007.06.004.
5. Gazi, E.; Dwyer, J.; Lockyer, N.P.; Gardner, P.; Shanks, J.H.; Roulson, J.; Hart, C.A.; Clarke, N.W.; Brown, M.D. Biomolecular Profiling of Metastatic Prostate Cancer Cells in Bone Marrow Tissue Using FTIR Microspectroscopy: A Pilot Study. *Anal. Bioanal. Chem.* **2007**, *387*, 1621–1631, doi:10.1007/s00216-006-1093-y.
6. Kacuráková, M. FT-IR Study of Plant Cell Wall Model Compounds: Pectic Polysaccharides and Hemicelluloses. *Carbohydr. Polym.* **2000**, *43*, 195–203, doi:10.1016/S0144-8617(00)00151-X.
7. Brézillon, S.; Untereiner, V.; Lovergne, L.; Tadeo, I.; Noguera, R.; Maquart, F.X.; Wegrowski, Y.; Sockalingum, G.D. Glycosaminoglycan Profiling in Different Cell Types Using Infrared Spectroscopy and Imaging. *Anal. Bioanal. Chem.* **2014**, *406*, 5795–5803, doi:10.1007/s00216-014-7994-2.
8. Mohamed, H.T.; Untereiner, V.; Cinque, G.; Ibrahim, S.A.; Götte, M.; Nguyen, N.Q.; Rivet, R.; Sockalingum, G.D.; Brézillon, S. Infrared Microspectroscopy and Imaging Analysis of Inflammatory and Non-Inflammatory Breast Cancer Cells and Their GAG Secretome. *Molecules* **2020**, *25*, 4300, doi:10.3390/molecules25184300.
9. Pan, N.C.; Pereira, H.C.B.; da Silva, M. de L.C.; Vasconcelos, A.F.D.; Celligoi, M.A.P.C. Improvement Production of Hyaluronic Acid by Streptococcus Zooepidemicus in Sugarcane Molasses. *Appl. Biochem. Biotechnol.* **2017**, *182*, 276–293, doi:10.1007/s12010-016-2326-y.
10. Derenne, A.; Derfoufi, K.-M.; Cowper, B.; Delporte, C.; Goormaghtigh, E. FTIR Spectroscopy as an Analytical Tool to Compare Glycosylation in Therapeutic Monoclonal Antibodies. *Anal. Chim. Acta* **2020**, *1112*, 62–71, doi:10.1016/j.aca.2020.03.038.
11. Kirschbaum, C.; Greis, K.; Mucha, E.; Kain, L.; Deng, S.; Zappe, A.; Gewinner, S.; Schöllkopf, W.; von Helden, G.; Meijer, G.; et al. Unravelling the Structural Complexity of Glycolipids with Cryogenic Infrared Spectroscopy. *Nat. Commun.* **2021**, *12*, 1201, doi:10.1038/s41467-021-21480-1.
12. Banyay, M.; Sarkar, M.; Gräslund, A. A Library of IR Bands of Nucleic Acids in Solution. *Biophys. Chem.* **2003**, *104*, 477–488, doi:10.1016/s0301-4622(03)00035-8.
13. Synytsya, A. Fourier Transform Raman and Infrared Spectroscopy of Pectins. *Carbohydr. Polym.* **2003**, *54*, 97–106, doi:10.1016/S0144-8617(03)00158-9.
14. Foster, A.B.; Martlew, E.F.; Stacey, M.; Taylor, P.J.M.; Webber, J.M. 236. Amino-Sugars and Related Compounds. Part VIII. Some Properties of 2-Deoxy-2-Sulphoamino-D-Glucose, Heparin, and Related Substances. *J. Chem. Soc. Resumed* **1961**, 1204, doi:10.1039/jr9610001204.
15. Parker, F.S. *Applications of Infrared Spectroscopy in Biochemistry, Biology, and Medicine*; Springer US: Boston, MA, 1971; ISBN 978-1-4684-1874-3.
